# Occupational Exposures and Neurodegenerative Diseases—A Systematic Literature Review and Meta-Analyses

**DOI:** 10.3390/ijerph16030337

**Published:** 2019-01-26

**Authors:** Lars-Gunnar Gunnarsson, Lennart Bodin

**Affiliations:** 1 Department of Occupational and Environmental Medicine, School of Medicine, Örebro University, 701 82 Örebro, Sweden; 2 Department of Statistics, Örebro University, 701 82 Örebro, Sweden; lennart.bodin@oru.se; 3 Institute of Environmental Medicine, Karolinska Institute, SE 177 77 Stockholm, Sweden

**Keywords:** epidemiology, metals, pesticides, electromagnetic fields, ALS, Parkinson’s disease, Alzheimer’s disease

## Abstract

*Objectives:* To carry out an integrated and stratified meta-analysis on occupational exposure to electromagnetic fields (EMFs), metals and pesticides and its effects on amyotrophic lateral sclerosis (ALS) and Parkinson’s and Alzheimer’s disease, and investigate the possibility of publication bias. *Methods*: In the current study, we updated our recently published meta-analyses on occupational exposures in relation to ALS, Alzheimer’s and Parkinson’s disease. Based on 66 original publications of good scientific epidemiological standard, according to the Meta-analysis of Observational Studies in Epidemiology (MOOSE) and the Grading of Recommendations, Assessment, Development and Evaluations (GRADE) guidelines, we analysed subgroups by carrying out stratified meta-analyses on publication year, statistical precision of the relative risk (RR) estimates, inspection of the funnel plots and test of bias. *Results*: Based on 19 studies the weighted RR for occupational exposure to EMFs was 1.26 (95% confidence interval (CI) 1.07–1.50) for ALS, 1.33 (95% CI 1.07–1.64) for Alzheimer’s disease and 1.02 (95% CI 0.83–1.26) for Parkinson’s disease. Thirty-one studies concerned occupational exposure to pesticides and the weighted RR was 1.35 (95% CI 1.02–1.79) for ALS, 1.50 (95% CI 0.98–2.29) for Alzheimer’s disease and 1.66 (95% CI 1.42–1.94) for Parkinson’s disease. Finally, 14 studies concerned occupational exposure to metals and only exposure to lead (five studies) involved an elevated risk for ALS or Parkinson’s disease and the weighted RR was 1.57 (95% CI 1.11–2.20). The weighted RR for all the non-lead exposures was 0.97 (95% CI 0.88–1.06). *Conclusions*: Exposure to pesticides increased the risk of getting the mentioned neurodegenerative diseases by at least 50%. Exposure to lead was only studied for ALS and Parkinson’s disease and involved 50% increased risk. Occupational exposure to EMFs seemed to involve some 10% increase in risk for ALS and Alzheimer’s disease only.

## 1. Background

Parkinson’s disease, amyotrophic lateral sclerosis (ALS) and Alzheimer’s disease are the most common neurodegenerative disorders. They primarily affect ageing individuals and are characterized by a steady progressive course because of increasing loss of specific neurons in the brain. Over the last two decades our understanding of the pathophysiological processes of neurodegeneration has grown extensively owing to huge progress in biochemical research. Targets in all three diseases are specific large molecules that are essential for ensuring normal function and survival of the neuron cell. There is a continuous turnover of these molecules and several enzymatic pathways are involved in their production and their degradation. With age, more and more damaged or misfolded defective molecules are stored in inclusion bodies (“garbage bags”) in and between neurons, thus enhancing degeneration of cells. The defective molecules also disturb the function of the neuron, leading to cell death. Promoters in the molecule-damaging process are hereditary insufficiencies as well as exposure to chemicals, all influencing the turnover of the target protein. However, the damaging process can also be influenced by stress and degree of metabolic activity in the cell, as well as other factors.

Several systematic literature reviews including meta-analyses have been published on epidemiological studies of occupational exposure and neurodegenerative diseases. The most frequently studied exposures have been pesticides [1,2,3,4,5,6,7], extremely low frequency electromagnetic fields (EMFs) [8,9,10,11,12,13] and metals [14,15]. These meta-analyses have included all cohort, case-control and register studies with appropriate exposure, but have not excluded any studies because of lacking scientific quality. They have not applied the guidelines proposed by the Meta-analysis of Observational Studies in Epidemiology (MOOSE) [16] and Grading of Recommendations, Assessment, Development and Evaluations (GRADE) [17,18] groups. The three systematic literature reviews [19,20,21] we have carried out and published, by contrast, include only studies of good scientific standard, according to the MOOSE and GRADE guidelines.

The aim of the present paper was to update our database, carry out integrated and stratified meta-analyses, based only on publications of good scientific standard, on neurodegenerative diseases in relation to main occupational exposures, and investigate the possibility of publication bias.

## 2. Methods

Using the same search criteria as in our previous meta-analyses [19,20,21] and the bibliographic search engines in PubMed and Embase we identified another three relevant studies [22,23,24] published in 2017. All relevant publications (those from our previous systematic literature reviews together with the new ones) were scrutinized according to MOOSE and GRADE guidelines. We assessed them according to Armon classification [25] using standardized protocols, see Supplementary Materials S1. None of the publications were classified as Armon class I, which requires an almost experimental design. Only publications with good scientific standard (Armon global score II or III) were used in our meta-analyses. All these studies fulfilling good scientific standards are presented in detailed tables in our recently published, disease-specific meta-analyses [19,20,21]. Articles not qualifying for score II–III either had serious weaknesses (Armon score IV) or should be ignored (Armon score V).

### Statistical Methods

Risk estimates from the selected studies are reported as relative risks (RRs), as the outcome is rare. For the purposes of this research, we considered odds ratio (OR) and hazard ratio (HR) equivalent to RR. Where a study reported unadjusted as well as multivariable-adjusted risk estimates, we only considered the adjusted estimates. Studies that reported stratified estimates for sex were treated as separate studies and the estimates of each were included. Where exposure was categorized into different levels the risk rate for the highest level was used. Some studies reported estimates for more than one neurodegenerative disorder and all are included in the summary.

We examined the fixed effects model as well as the random effects model by considering statistical heterogeneity. To this end, we used the *I*^2^ statistic and applied the recommended cut-offs of 25%, 50% and 75% degrees of heterogeneity. We stratified the data by study characteristics (gender, source of funding, exposure characteristics, previous vs. recent studies, low statistical precision studies vs. high precision studies), selected a priori, and used meta-regression to evaluate the significance of the stratification variable. Based on the *I*^2^ criterion and the meta-regression, a random effects model was found to be the most appropriate choice in all of our analyses; hence, the results are reported only with random effects estimates. The weights used for pooling the risk estimates were equal to the inverse-variance weighting. Pooled risk estimates are presented with 95% confidence intervals (CIs).

Publication bias was analysed by inspection of the funnel plot, in which the estimates of RR should be distributed symmetrically around the weighted RR unless the publication was affected by bias. The rank correlation test proposed by Begg and Mazumdar [26] was used to supplement the interpretation of the funnel plot. We investigated whether publication bias could be related to (i) publication year, where early studies of a more exploratory character were more likely to be published if they showed increased RRs, or (ii) the statistical precision of the estimated RR, where studies with less precise CIs for RR were more likely to be published if they indicated an increased RR. To accomplish the stratification of year of publication we used stratification with cut-off points around 2005 and 2006. For statistical precision, we used the standard error of ^e^log(RR), the essential factor that affects the width of the 95% CI for RR. The practical application was to use a value of 0.26 for this standard error, which rendered the two strata (low vs. high precision) to be approximately equal in numbers of estimated RRs for several of the comparisons. The width of the CI is dependent both on the total number of subjects in the study and on the number of cases with the specified diagnosis. We therefore use the phrases “estimate with high precision” and “estimate with low precision” rather than “study with high precision” and “study with low precision”. It is possible that the same study with more than one diagnosis analysed may have contributed to one estimate with high and another estimate with low precision.

Statistical analyses were conducted using procedures for different aspects of meta-analyses available in STATA software, version 15.2 (www.Stata.com, StataCorp, College Station, TX, USA), and described in articles from the STATA journal [27]. A procedure particularly useful as a starting point for analysing publication bias was Metacum which starts by ordering the RR from the analysed studies according to a chosen criterion such as year of publication or width of the confidence interval, i.e., the statistical precision. RR is then updated starting with the first RR in the ordered sequence and recalculating a cumulated RR as more studies are added for analysis according to the applied ordering. The Metacum graph gives an insight whether the cumulated RR is constant, decreasing or increasing over time (or precision) which is useful information when evaluating a possible publication bias.

## 3. Results

### 3.1. Exposure to Low Frequency Electromagnetic Fields

**Figure 1:**
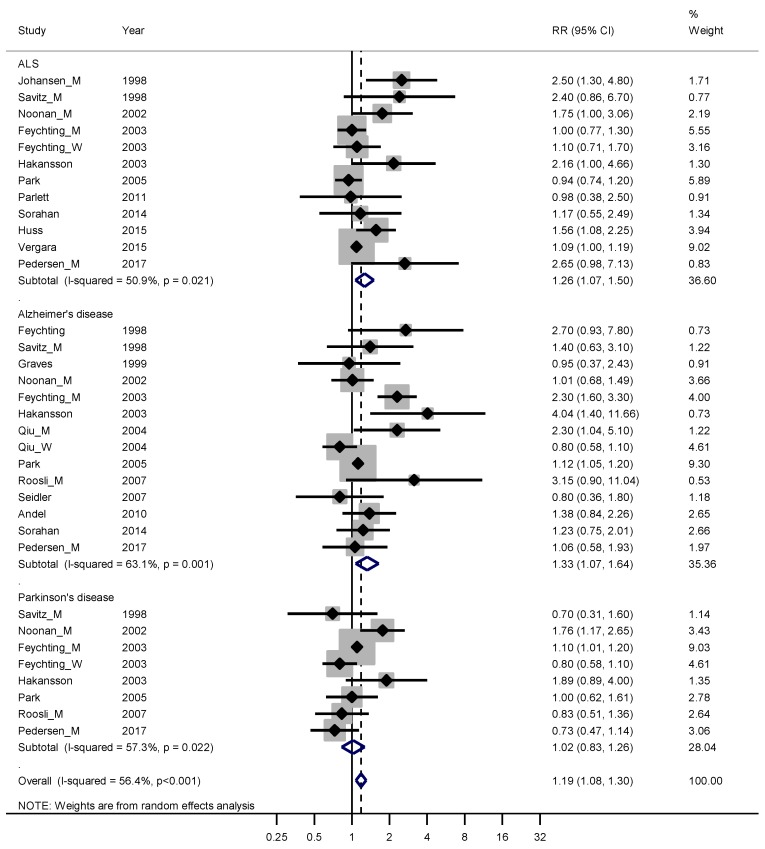
Forest plot for studies assessing the association between amyotrophic lateral sclerosis (ALS), Alzheimer’s disease and Parkinson’s disease and occupational exposure to electromagnetic fields (EMFs). Results for men only are indicated by M, for women by W; otherwise the results concern both sexes. Random effect models were used, with stratification by diagnosis. Heterogeneity was tested by the *I*^2^ statistic (*I*-squared), with *p* < 0.05 indicating rejection of homogeneity. CI = confidence interval; RR = relative risk.

Sixteen studies fulfilled good scientific standards (Armon class II–III) and contributed data to meta-analyses of risk rates regarding exposure to EMFs: eleven studies on ALS [22,28,29,30,31,32,33,34,35,36,37], 13 on Alzheimer’s disease [2,6,7,8,9,10,11,12,13,14,15,16,17,18,19,20,21,22,25,26,27,28,29,30,31,32,33,35,38,39,40,41,42,43] and seven on Parkinson’s disease [22,29,30,31,32,33,41]. Figure 1 shows that the weighed RRs for ALS and Alzheimer’s disease were slightly elevated, 1.26 (95% CI 1.07–1.50) and 1.33 (95% CI 1.07–1.64), respectively. The weighted RR was not elevated for Parkinson’s disease. Funnel plots and Begg’s test for the respective disease indicated slight publication bias for ALS and Alzheimer’s disease but not for Parkinson’s disease.

**Figure 2:**
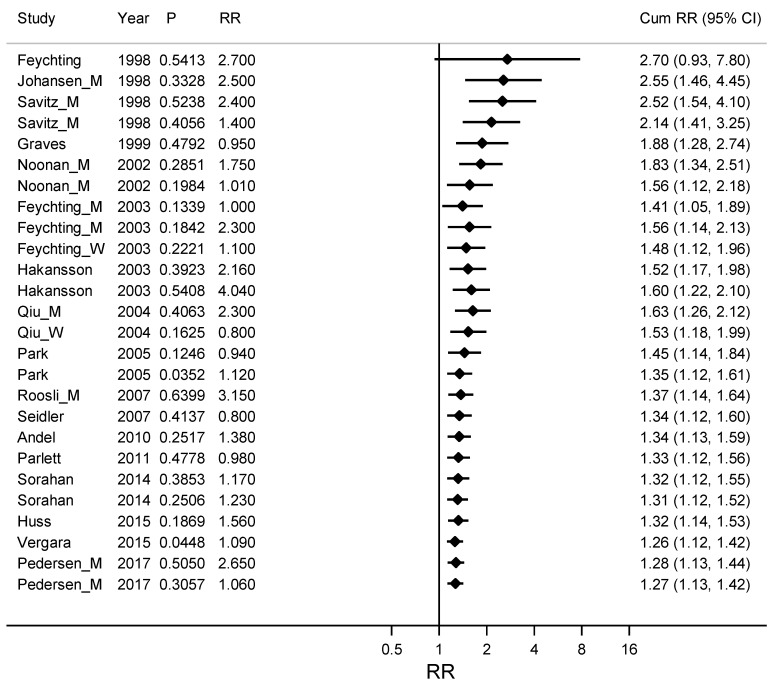
Cumulative meta-analyses by ordered study year from early to late years on the association between ALS (amyotrophic lateral sclerosis) and Alzheimer’s disease together and occupational exposure to electromagnetic fields (EMFs) with the pooled estimate (Cum RR) updated for every new study. Column P gives a measure of the precision for the confidence interval for RR, where large P stands for a wide confidence interval and small P for narrow and more precise confidence interval. RR is the Relative Risk for the study included in the Cum RR.

To test the hypothesis that publication bias had a higher influence on earlier publications we made a Metacum on ALS and Alzheimer’s disease together, stratified by year of publication (See Figure 2). The weighted RR for the ten publications from before 2005 was 1.53 (95% CI 1.18–1.99), compared with 1.12 (95% CI 1.04–1.20) for nine publications from 2005 or later.

**Figure 3:**
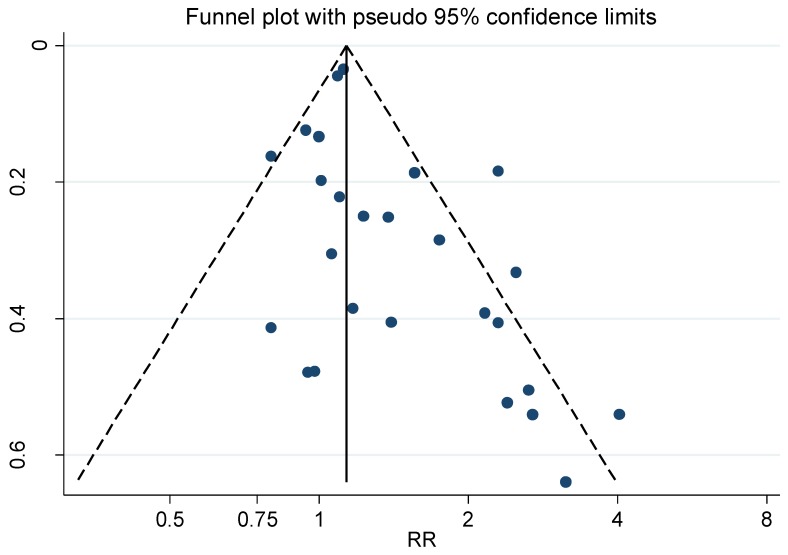
Funnel plot for the relative risk (RR) estimates from all studies of the association between amyotrophic lateral sclerosis (ALS) and Alzheimer’s disease together and occupational exposure to electromagnetic fields (EMFs) as shown in Figure 1. Begg’s test, *p* = 0.023.

The corresponding funnel plots of studies on ALS and Alzheimer’s disease together (see Figure 3) showed an evident publication bias.

**Figure 4:**
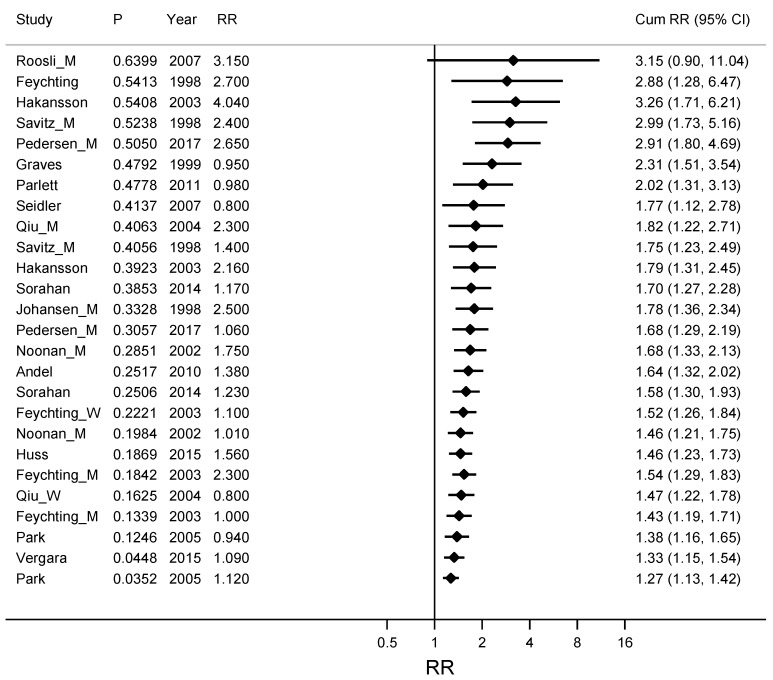
Cumulative meta-analyses for studies ordered by precision of the confidence interval for RR, from low precision with wide confidence intervals for RR to studies with high precision and narrow confidence intervals. The association between ALS (amyotrophic lateral sclerosis) and Alzheimer’s disease together and occupational exposure to electromagnetic fields (EMFs) is described. The pooled estimate (Cum RR) is updated for every new study. Column P gives a measure of the precision for the confidence interval for RR, where large P stands for a wide confidence interval and small P stands for a narrow and more precise confidence interval. RR is the Relative Risk for the study included in the Cum RR.

Estimates from the studies on ALS and Alzheimer’s disease were grouped into estimates with narrow CIs (high precision estimates) and estimates with wider CIs (low precision estimates) and a Metacum based on an ordering by precision is shown in Figure 4. The weighted RR for these eight studies with high precision estimates [30,31,33,35,36,37,40,43] was 1.14 (95% CI 1.01–1.27) compared with RR 1.68 (95% CI 1.33–2.13) for the twelve studies with low precision estimates [22,28,29,30,32,34,35,38,39,40,41,42].

### 3.2. Exposure to Pesticides

**Figure 5:**
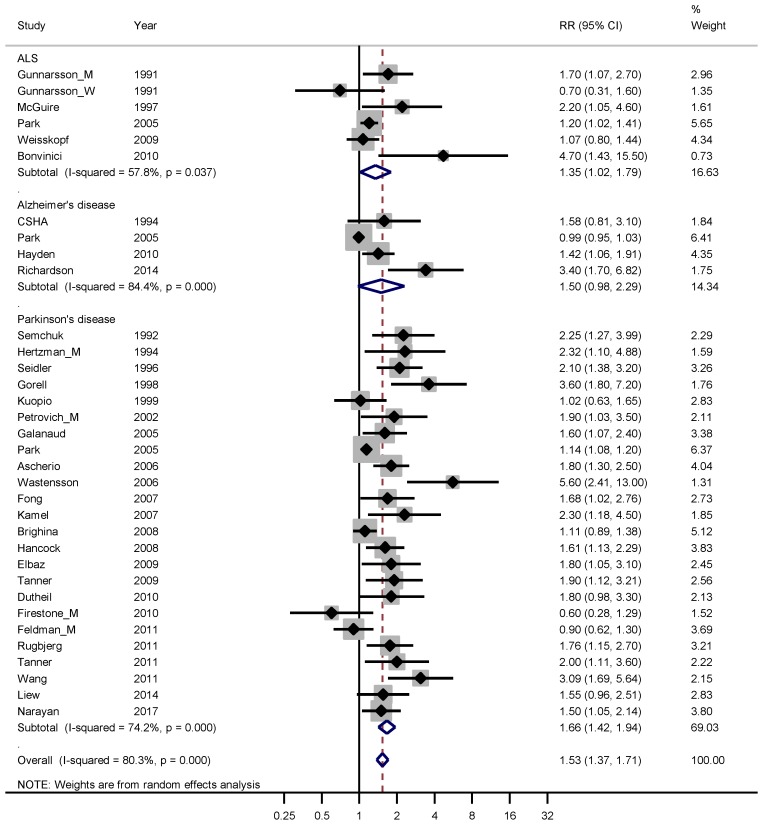
Forest plot for studies assessing the association between amyotrophic lateral sclerosis (ALS), Alzheimer’s disease and Parkinson’s disease and occupational exposure to pesticides. Results for men only are indicated by M, for women by W; otherwise the results concern both sexes. Random effect models were used, with stratification by diagnosis. Heterogeneity was tested by the *I*^2^ statistic (*I*-squared), with *p* < 0.05 indicating rejection of homogeneity. CI = confidence interval; RR = relative risk.

Thirty-one studies fulfilling good scientific standards (Armon class II–III) contributed data to meta-analyses of risk rates for pesticide exposure: five studies on ALS [33,44,45,46,47], four on Alzheimer’s disease [33,48,49,50] and 24 on Parkinson’s disease [23,33,51,52,53,54,55,56,57,58,59,60,61,62,63,64,65,66,67,68,69,70,71,72]. Figure 5 shows an increased overall risk of 50% for contracting these neurodegenerative diseases following exposure to pesticides.

**Figure 6:**
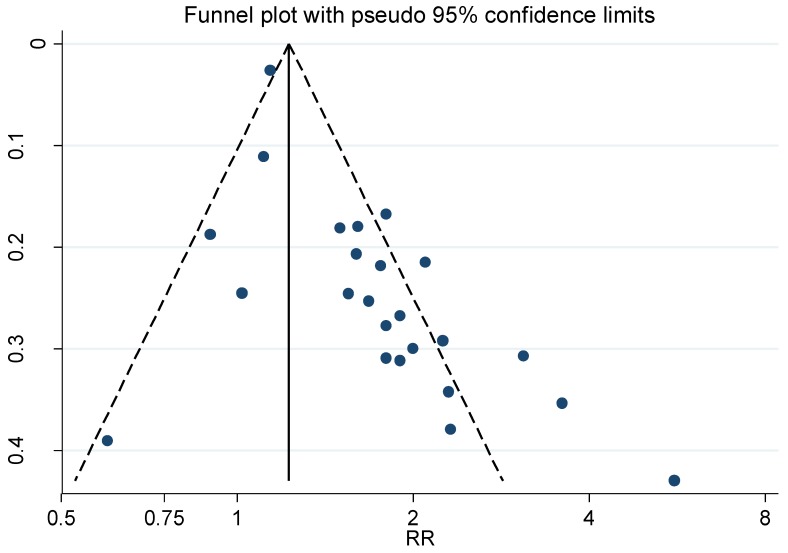
Funnel plot for the relative risk (RR) estimates from studies of the association between Parkinson’s disease and occupational exposure to Pesticides, Begg’s test = 0.026.

The weighted RR for ALS, Alzheimer’s and Parkinson’s disease was 1.35 (95% CI 1.02–1.79), 1.50 (95% CI 0.98–2.29) and 1.66 (95% CI 1.42–1.94), respectively. The weighted funnel plot (see Figure 6) showed an evident publication bias also indicated by Begg’s test (*p* = 0.060).

Only analysing the 24 out of 31 studies concerning Parkinson’s disease resulted in even more marked publication bias, Begg’s test *p* = 0.026. The weighted RR for the six Parkinson’s studies published before 2005 was 1.98 (95% CI 1.41–2.77), compared with 1.57 (95% CI 1.33–1.85) for the 18 studies published in or after 2005. The estimates from these studies were also dichotomized according to precision. The twelve estimates with the highest precision, in studies [23,33,53,55,57,58,61,62,63,68,71,72], had a weighted RR of 1.39 (95% CI 1.20–1.62). For the low precision estimates, in studies [51,52,54,56,59,60,64,65,66,67,69,70], the corresponding RR was 2.14 (95% CI 1.66–2.74).

### 3.3. Exposure to Metals

**Figure 7:**
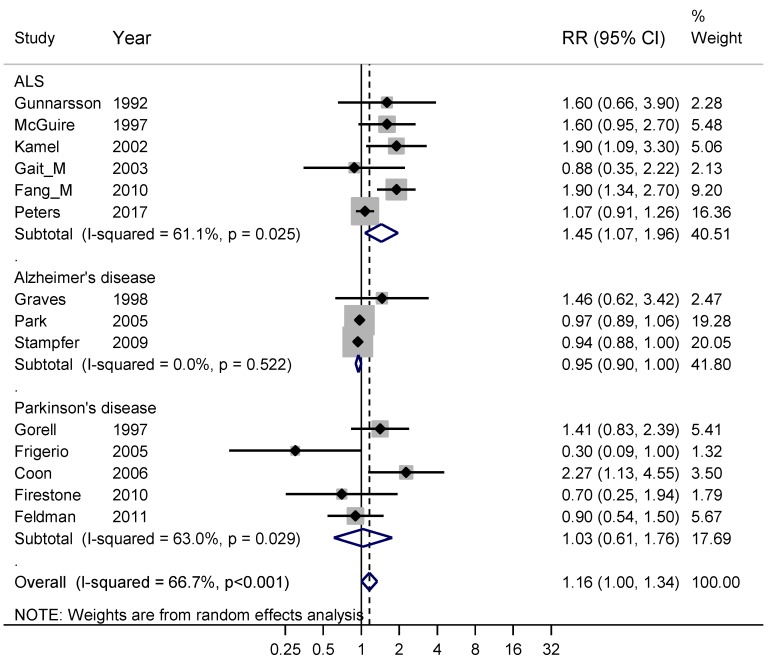
Forest plot for studies assessing the association between amyotrophic lateral sclerosis (ALS), Alzheimer’s disease and Parkinson’s disease and occupational exposure to metals. Results for men only are indicated by M, otherwise the results concern both sexes. Random effect models were used, with stratification by diagnosis. Heterogeneity was tested by the *I*^2^ statistic (*I*-squared), with *p* < 0.05 indicating rejection of homogeneity. CI = confidence interval; RR = relative risk.

Fourteen studies fulfilling good scientific standards (Armon class II–III) contributed data to meta-analyses of risk rates regarding exposure to metals: six studies on ALS [24,45,73,74,75,76], three on Alzheimer’s disease [33,77,78] and five on Parkinson’s disease [67,71,79,80,81]. Figure 7 shows that exposure to metals only entailed an increased risk for ALS, with a weighted RR of 1.45 (95% CI 1.07–1.96). The risk estimate 1.16 based on all three diagnoses relates to exposure to a mixture of metals. However, sub-analysis of the five studies [24,74,76,79,81] with lead exposure showed an RR of 1.57 (95% CI 1.11–2.20). The studies that did not involve lead were one [77] related to exposure to aluminum, three [45,75,80] to a mixture of metals and five [33,67,71,73,78] to welding and the supplementary sub-analysis for all these non-lead exposures gave RR 0.97 (95% CI 0.88–1.06). A funnel plot of all studies in Figure 7 indicated slight publication bias for all three diagnoses together, although not supported by Begg’s test, *p* = 0.70.

Exposure to welding involves exposure to both a mixture of metals and EMFs. Six studies of high scientific standard reported risk estimates for ALS [31,33,73,78,82,83], giving a weighted RR of 0.95 (95% CI 0.70–1.29). Eight studies of high scientific standard concerned Parkinson’s disease [33,64,67,71,78,84,85,86] and the weighted RR was 0.85 (95% CI 0.82–0.89). Separate funnel plots for ALS and Parkinson’s disease did not indicate publication bias. Analysing both diseases together gave a weighed RR of 1.00 (95% CI 0.73–1.38) for the five studies published before 2005 compared with 0.85 (95% CI 0.77–0.94) for the nine studies published later.

## 4. Discussion

Recently we have presented three meta-analyses on occupational exposures in relation to ALS, and Alzheimer’s or Parkinson’s disease [19,20,21], based on studies with a good scientific standard, according to Armon [25]. In the present study, we focused on exposure to EMFs, pesticides and metals and, using different stratifications, have tried to eliminate the effects of publication bias. Our results show that exposure to pesticides brought about at least 50% increased risk for contracting these neurodegenerative diseases. Exposure to lead involved at least a significant risk for ALS and Parkinson’s disease. Numerous previous studies have indicated that exposure to EMFs involves risk for neurodegenerative diseases. Our analyses indicate that the proposed risk could in part be attributed to publication bias.

### 4.1. Exposure to Electromagnetic Fields

Elevated weighted RRs were obtained for only ALS and Alzheimer’s disease. In almost all the included studies, occupational exposure to low-frequency EMFs was assessed from a validated job exposure matrix applied to job titles according to census registers or other registers. Questionnaires and/or interviews were used in two studies only [39,40]. Studies fulfilling the highest scientific standards for quality, i.e., Armon class II [19], were found in both diseases [36,40,43].

The weighted RR from studies published in 2005 or later was 1.12 (95% CI 1.04–1.20) and there was no publication bias for this period. The weighted risk estimate for earlier publications (RR = 1.53) was hampered by an evident publication bias indicating that the true risk-RR was closer to 1.12. This risk estimate also corresponds to the estimate derived from the high-precision studies where the weighted RR was 1.14.

Below we discuss four systematic reviews and meta-analyses on exposure to EMFs and neurodegenerative diseases. Only some of the included studies fulfil good scientific standards, according to Armon [25] (class II–III), which was the criterion we used for inclusion in our meta-analyses. We have scrutinized all the studies and, based on the Armon classification, have classified the remaining studies as class IV–V, thus indicating that they do not fulfil good scientific standards.

Two meta-analyses concerned Alzheimer’s disease and occupational exposure to EMFs. The earlier of these [8] included twelve studies, nine of which [29,30,31,32,38,39,40,87,88] had a high scientific standard. The weighted RR was 2.03 (95% CI 1.38–3.00) for the case-control studies and 1.62 (95% CI 1.16–2.27) for the cohort studies. The other meta-analysis [12] included 20 studies and the weighted RR was 1.63 (95% CI 1.35–1.96). Fourteen out of these studies [22,29,30,31,32,33,35,38,39,40,41,42,43,87,88] were of a good scientific standard.

Three meta-analyses concerned ALS and occupational exposure to EMFs [9,11,13]. The first [9] included 17 studies, 13 of which [29,30,31,32,33,34,41,44,73,87,88,89] had a high scientific standard and provided a weighted RR of 1.29 (95% CI 1.02–1.62). The second [11] included nine publications, eight of which [30,32,33,34,35,41,87,88] fulfilled good scientific standards and the weighted RR was estimated at 1.6 (95% CI 0.59–5.34). The third [13] included 20 studies, 14 of which [29,30,31,32,33,34,35,36,37,41,44,73,83,87] were of a good scientific standard. The weighted RR was 1.14 (95% CI 1.00–1.30). Finally, one meta-analysis [10] provided separate analysis for the degenerative diseases and different stratifications with regard to study design and exposure assessment.

Taken together, the weighted risk estimates in these meta-analyses on EMF were-based both on studies with a high scientific standard, according to Armon [25], and research that was hampered by different types of biases and weaknesses, which was especially apparent in studies published before 2005. This explains why the weighted RR found in our stratified meta-analyses was in the lower range, indicating only about 10% elevated risk for ALS or Alzheimer’s disease after occupational exposure to low-frequency EMFs.

### 4.2. Exposure to Pesticides

The highest impact on the weighted RR for Parkinson’s disease came from the biggest study which was based on the US mortality register. Here the exposure was assessed from a validated job exposure matrix related to the deceased persons’ main occupation, according to census data [33]. In all the other 23 studies, information on pesticide exposure was obtained by questionnaires and/or interviews. Although the design of that study was different from the other studies, another three big studies [55,62,71] likewise showed RRs below the weighted mean. Studies fulfilling the highest scientific standards for quality, i.e., Armon class II [19], were found both in the groups of big studies [53,58,62,72] and among the smaller studies [51,56,65,66,69]. The twelve studies with lower precision had a weighted RR of 2.14, compared with 1.39 for the twelve studies with higher precision. This might be explained by publication bias where studies with high risk estimates are more often accepted for publication. Furthermore, studies published before 2005 had a higher weighted RR than studies published later (1.98 compared to 1.57). This might be explained by better regulations of pesticide exposure on the labor market.

There were too few studies published on pesticides in relation to ALS and Alzheimer’s disease to discuss the estimated weighted risk estimates for these diseases.

Eight systematic reviews and meta-analyses have been published regarding occupational exposure to pesticides and neurodegenerative diseases. Only some of the included studies fulfill good scientific standards and all remaining studies were classified as Armon class IV–V, indicating inability to fulfill good scientific standards.

Four systematic reviews and meta-analyses have been published on Parkinson’s disease and exposure to pesticides. The oldest study [1] included 19 publications with a weighted RR of 1.95 (95% CI 1.49–2.53). Only four of the included studies [51,52,53,54] were of good scientific standard. The second meta-analysis [2] included twelve publications giving an weighted RR 1.28 (95% CI 1.03–1.59), and three of these [56,58,59] fulfilled good scientific standards. The third meta-analysis [5] included 28 publications giving a weighted RR of 1.42 (95% CI 1.32–1.52), seven studies of which [53,55,57,58,60,62,63] had good scientific standards. The fourth study [7] presents a dose-response meta-analysis based on ten publications, with five [53,56,60,63,65] fulfilling good scientific standards.

Exposure to pesticides and ALS was studied in three systematic reviews and meta-analyses. The first meta-analysis [3] included ten publications, five of which [45,46,47,73,90] met the criteria for good scientific standards. The weighted RR was 1.88 (95% CI 1.36–2.61). The second study [4] included eight publications, five of which [45,46,47,73,90] had good scientific standards. The weighted RR was 2.2 (95% CI 1.5–3.3). The third study [11] used exacting inclusion criteria, and therefore only two [4,45] out of 17 studies fulfilled stipulated criteria for scientific quality, and the weighted RR was 1.01 (95% CI 0.75–1.58).

Only one analysis [6] presents a systematic review and meta-analysis on Alzheimer’s disease and exposure to pesticides. Based on seven studies, it reports a weighted RR of 1.34 (95% CI 1.08–1.67). Two of the studies [49,91] fulfilled good scientific standards, according to Armon [25].

Since only a minority of the studies included in the above meta-analyses were of good scientific standard, according to Armon [25], the presented risk estimates should be interpreted with great caution. However, the estimates are in the same order as the weighted RR we found in our stratified meta-analyses. Therefore it seems safe to conclude that occupational exposure to pesticides brings about at least 50% increased risk for contracting a neurodegenerative disease.

### 4.3. Exposure to Metals

In the studied populations, exposure to lead came out to be the significant exposure involving a weighed RR 1.57 for ALS and Parkinson’s disease and this risk estimate was not hampered by publication bias. Regarding Alzheimer’s disease we have no epidemiological data on lead exposure and can thus not evaluate if lead also involves a risk for this disease. One study present an increased risk after exposure to aluminum [77]. Exposure to a mixture of metals (including welding) did not seem to involve risk in any of these degenerative diseases.

One systematic review and meta-analysis has been published regarding ALS and occupational exposure to metals [11]. It identified only two studies [45,75] and were of high scientific standard. Another systematic review and meta-analysis has been published regarding ALS and occupational exposure to lead [15]. Based on nine studies it estimated the weighted RR at 1.81 (95% CI 1.39–2.36) but only two of the studies [45,74] were of good scientific standard, according to Armon [25]. One systematic review and meta-analysis has been published regarding Parkinson’s disease and occupational exposure to welding [14]. Based on nine studies, five of which had a high scientific standard, the weighted RR was 0.86 (95% CI 0.80–0.92).

The results from the abovementioned meta-analyses and our own analysis all confirm that there seems to be about 50% increased risk for ALS and Parkinson’s disease after occupational exposure to lead. Exposure to a mixture of metals or welding does not seem to involve an increase in risk.

### 4.4. Strengths and Limitations

All previously published meta-analyses on the neurodegenerative diseases ALS, Alzheimer’s disease and Parkinson’s disease are based on all publications on the topic that were identified, irrespective of the scientific quality of the study design. One strength of our study is that our meta-analyses were based on a systematic literature review that included only studies of a high scientific standard. Based on the detailed checklist proposed by Armon [25] we used an elaborate protocol for scrutinizing publications [20]. Another strength of our meta-analyses is that we focused heavily on finding all possible sources of bias, using stratification of data with regard to possible confounders. For evaluation of possible publication bias, we used several methods including funnel plots, specific tests and stratification by publication year as well as precision of risk estimates.

General limitations of meta-analyses are that the calculations can only be based on published data and will reflect any inherent weaknesses of design in the studies included. Another limitation is that published epidemiologic studies only present aggregated data on exposure based on questionnaires, interviews, a validated Job Exposure Matrix (associated with job titles) or a work place cohort with specified exposures. Furthermore, the dosage of exposure is not treated uniformly. In some studies exposure is graded Yes or No, in others the dosage is graded into 3–4 levels (but not uniformly between studies). Based on this and other heterogeneities in exposure we selected risk estimates covering medium or high levels and also gave priority to many years of exposure instead of peak exposures. The risk estimates given should thus be interpreted as averages for recurrent occupational exposures. However, according to the decision protocol (see Appendix) we meticulously have scrutinized the quality of exposure data in every included publication.

## 5. Conclusions

Our meta-analyses showed that exposure to pesticides carried at least a 50% increased risk for contracting Parkinson’s disease, Alzheimer’s disease or ALS, ranked in order of risk. Exposure to lead seemed to involve at least 50% increased risk for getting ALS or Parkinson’s disease, while non-lead exposures did not seem to involve risk. Occupational exposure to EMFs seemed to involve some 10% increase in risk for ALS and Alzheimer’s disease, but no such indication of risk was found for Parkinson’s disease.

## Supplementary Materials

The following are available online at http://www.mdpi.com/1660-4601/16/3/337/s1, S1: Decision protocol for grading studies regarding scientific epidemiologic standard.

## References

[1] Priyadarshi A., Khuder S.A., Schaub E.A., Shrivastava S. (2000). A meta-analysis of Parkinson’s disease and exposure to pesticides. Neurotoxicology.

[2] Van Maele-Fabry G., Hoet P., Vilain F., Lison D. (2012). Occupational exposure to pesticides and Parkinson’s disease: A systematic review and meta-analysis of cohort studies. Environ. Int..

[3] Malek A.M., Barchowsky A., Bowser R., Youk A., Talbott E.O. (2012). Pesticide exposure as a risk factor for amyotrophic lateral sclerosis: A meta-analysis of epidemiological studies: Pesticide exposure as a risk factor for ALS. Environ. Res..

[4] Kamel F., Umbach D.M., Bedlack R.S., Richards M., Watson M., Alavanja M.C., Blair A., Hoppin J.A., Schmidt S., Sandler D.P. (2012). Pesticide exposure and amyotrophic lateral sclerosis. Neurotoxicology.

[5] Allen M.T., Levy L.S. (2013). Parkinson’s disease and pesticide exposure—A new assessment. Crit. Rev. Toxicol..

[6] Yan D., Zhang Y., Liu L., Yan H. (2016). Pesticide exposure and risk of Alzheimer’s disease: A systematic review and meta-analysis. Sci. Rep..

[7] Yan D., Zhang Y., Liu L., Shi N., Yan H. (2018). Pesticide exposure and risk of Parkinson’s disease: Dose-response meta-analysis of observational studies. Regul. Toxicol. Pharmacol..

[8] Garcia A.M., Sisternas A., Hoyos S.P. (2008). Occupational exposure to extremely low frequency electric and magnetic fields and Alzheimer disease: A meta-analysis. Int. J. Epidemiol..

[9] Zhou H., Chen G., Chen C., Yu Y., Xu Z. (2012). Association between extremely low-frequency electromagnetic fields occupations and amyotrophic lateral sclerosis: A meta-analysis. PLoS ONE.

[10] Vergara X., Kheifets L., Greenland S., Oksuzyan S., Cho Y.S., Mezei G. (2013). Occupational exposure to extremely low-frequency magnetic fields and neurodegenerative disease: A meta-analysis. J. Occup. Environ. Med..

[11] Capozzella A., Sacco C., Chighine A., Loreti B., Scala B., Casale T., Sinibaldi F., Tomei G., Giubilati R., Tomei F. (2014). Work related etiology of amyotrophic lateral sclerosis (ALS): A meta-analysis. Ann. Ig..

[12] Jalilian H., Teshnizi S.H., Roosli M., Neghab M. (2017). Occupational exposure to extremely low frequency magnetic fields and risk of Alzheimer disease: A systematic review and meta-analysis. Neurotoxicology.

[13] Huss A., Peters S., Vermeulen R. (2018). Occupational exposure to extremely low-frequency magnetic fields and the risk of ALS: A systematic review and meta-analysis. Bioelectromagnetics.

[14] Mortimer J.A., Borenstein A.R., Nelson L.M. (2012). Associations of welding and manganese exposure with Parkinson disease: Review and meta-analysis. Neurology.

[15] Wang M.D., Gomes J., Cashman N.R., Little J., Krewski D. (2014). A meta-analysis of observational studies of the association between chronic occupational exposure to lead and amyotrophic lateral sclerosis. J. Occup. Environ. Med..

[16] Stroup D.F., Berlin J.A., Morton S.C., Olkin I., Williamson G.D., Rennie D., Moher D., Becker B.J., Sipe T.A., Thacker S.B. (2000). Meta-analysis of observational studies in epidemiology: A proposal for reporting. Meta-analysis of Observational Studies in Epidemiology (MOOSE) group. JAMA.

[17] Guyatt G.H., Oxman A.D., Vist G., Kunz R., Brozek J., Alonso-Coello P., Montori V., Akl E.A., Djulbegovic B., Falck-Ytter Y. (2011). GRADE guidelines: 4. Rating the quality of evidence—Study limitations (risk of bias). J. Clin. Epidemiol..

[18] Guyatt G.H., Oxman A.D., Sultan S., Glasziou P., Akl E.A., Alonso-Coello P., Atkins D., Kunz R., Brozek J., Montori V. (2011). GRADE guidelines: 9. Rating up the quality of evidence. J. Clin. Epidemiol..

[19] Gunnarsson L.G., Bodin L. (2017). Parkinson’s disease and occupational exposures: A systematic literature review and meta-analyses. Scand. J. Work Environ. Health.

[20] Gunnarsson L.G., Bodin L. (2018). Amyotrophic Lateral Sclerosis and Occupational Exposures: A Systematic Literature Review and Meta-Analyses. Int. J. Environ. Res. Public Health.

[21] Gunnarsson L.G., Bodin L. Alzheimer’s Disease and Occupational Exposures: A Systematic Literature Review and Meta-Analyses. Alzheimer’s Disease & Treatment.

[22] Pedersen C., Poulsen A.H., Rod N.H., Frei P., Hansen J., Grell K., Raaschou-Nielsen O., Schuz J., Johansen C. (2017). Occupational exposure to extremely low-frequency magnetic fields and risk for central nervous system disease: An update of a Danish cohort study among utility workers. Int. Arch. Occup. Environ. Health.

[23] Narayan S., Liew Z., Bronstein J.M., Ritz B. (2017). Occupational pesticide use and Parkinson’s disease in the Parkinson Environment Gene (PEG) study. Environ. Int..

[24] Peters T.L., Kamel F., Lundholm C., Feychting M., Weibull C.E., Sandler D.P., Wiebert P., Sparen P., Ye W., Fang F. (2017). Occupational exposures and the risk of amyotrophic lateral sclerosis. Occup. Environ. Med..

[25] Armon C. (2003). An evidence-based medicine approach to the evaluation of the role of exogenous risk factors in sporadic amyotrophic lateral sclerosis. Neuroepidemiology.

[26] Begg C.B., Mazumdar M. (1994). Operating characteristics of a rank correlation test for publication bias. Biometrics.

[27] Palmer T.M., Sterne J.A.C., Newton H.J., Cox N.J. (2016). Meta-Analysis in Stata: An Updated Collection from the Stata Journal.

[28] Johansen C., Olsen J.H. (1998). Mortality from amyotrophic lateral sclerosis, other chronic disorders, and electric shocks among utility workers. Am. J. Epidemiol..

[29] Savitz D.A., Checkoway H., Loomis D.P. (1998). Magnetic field exposure and neurodegenerative disease mortality among electric utility workers. Epidemiology.

[30] Noonan C.W., Reif J.S., Yost M., Touchstone J. (2002). Occupational exposure to magnetic fields in case-referent studies of neurodegenerative diseases. Scand. J. Work Environ. Health.

[31] Feychting M., Jonsson F., Pedersen N.L., Ahlbom A. (2003). Occupational magnetic field exposure and neurodegenerative disease. Epidemiology.

[32] Hakansson N., Gustavsson P., Johansen C., Floderus B. (2003). Neurodegenerative diseases in welders and other workers exposed to high levels of magnetic fields. Epidemiology.

[33] Park R.M., Schulte P.A., Bowman J.D., Walker J.T., Bondy S.C., Yost M.G., Touchstone J.A., Dosemeci M. (2005). Potential occupational risks for neurodegenerative diseases. Am. J. Ind. Med..

[34] Parlett L.E., Bowman J.D., van Wijngaarden E. (2011). Evaluation of occupational exposure to magnetic fields and motor neuron disease mortality in a population-based cohort. J. Occup. Environ. Med..

[35] Sorahan T., Mohammed N. (2014). Neurodegenerative disease and magnetic field exposure in UK electricity supply workers. Occup. Med. (Lond.).

[36] Huss A., Spoerri A., Egger M., Kromhout H., Vermeulen R. (2015). Occupational exposure to magnetic fields and electric shocks and risk of ALS: The Swiss National Cohort. Amyotroph. Lateral Scler. Frontotemporal Degener..

[37] Vergara X., Mezei G., Kheifets L. (2015). Case-control study of occupational exposure to electric shocks and magnetic fields and mortality from amyotrophic lateral sclerosis in the US, 1991–1999. J. Expo. Sci. Environ. Epidemiol..

[38] Feychting M., Pedersen N.L., Svedberg P., Floderus B., Gatz M. (1998). Dementia and occupational exposure to magnetic fields. Scand. J. Work Environ. Health.

[39] Graves A.B., Rosner D., Echeverria D., Yost M., Larson E.B. (1999). Occupational exposure to electromagnetic fields and Alzheimer disease. Alzheimer Dis. Assoc. Disord..

[40] Qiu C., Fratiglioni L., Karp A., Winblad B., Bellander T. (2004). Occupational exposure to electromagnetic fields and risk of Alzheimer’s disease. Epidemiology.

[41] Roosli M., Lortscher M., Egger M., Pfluger D., Schreier N., Lortscher E., Locher P., Spoerri A., Minder C. (2007). Mortality from neurodegenerative disease and exposure to extremely low-frequency magnetic fields: 31 years of observations on Swiss railway employees. Neuroepidemiology.

[42] Seidler A., Geller P., Nienhaus A., Bernhardt T., Ruppe I., Eggert S., Hietanen M., Kauppinen T., Frolich L. (2007). Occupational exposure to low frequency magnetic fields and dementia: A case-control study. Occup. Environ. Med..

[43] Andel R., Crowe M., Feychting M., Pedersen N.L., Fratiglioni L., Johansson B., Gatz M. (2010). Work-related exposure to extremely low-frequency magnetic fields and dementia: Results from the population-based study of dementia in Swedish twins. J. Gerontol. A Biol. Sci. Med. Sci..

[44] Gunnarsson L.G., Lindberg G., Soderfeldt B., Axelson O. (1991). Amyotrophic lateral sclerosis in Sweden in relation to occupation. Acta Neurol. Scand..

[45] McGuire V., Longstreth W.T., Nelson L.M., Koepsell T.D., Checkoway H., Morgan M.S., van Belle G. (1997). Occupational exposures and amyotrophic lateral sclerosis. A population-based case-control study. Am. J. Epidemiol..

[46] Weisskopf M.G., Morozova N., O’Reilly E.J., McCullough M.L., Calle E.E., Thun M.J., Ascherio A. (2009). Prospective study of chemical exposures and amyotrophic lateral sclerosis. J. Neurol. Neurosurg. Psychiatry.

[47] Bonvicini F., Marcello N., Mandrioli J., Pietrini V., Vinceti M. (2010). Exposure to pesticides and risk of amyotrophic lateral sclerosis: A population-based case-control study. Ann. Ist. Super. Sanita.

[48] The Canadian Study of Health and Aging (1994). Risk factors for Alzheimer’s disease in Canada. Neurology.

[49] Hayden K.M., Norton M.C., Darcey D., Ostbye T., Zandi P.P., Breitner J.C., Welsh-Bohmer K.A. (2010). Occupational exposure to pesticides increases the risk of incident AD: The Cache County study. Neurology.

[50] Richardson J.R., Roy A., Shalat S.L., von Stein R.T., Hossain M.M., Buckley B., Gearing M., Levey A.I., German D.C. (2014). Elevated serum pesticide levels and risk for Alzheimer disease. JAMA Neurol..

[51] Semchuk K.M., Love E.J., Lee R.G. (1992). Parkinson’s disease and exposure to agricultural work and pesticide chemicals. Neurology.

[52] Hertzman C., Wiens M., Snow B., Kelly S., Calne D. (1994). A case-control study of Parkinson’s disease in a horticultural region of British Columbia. Mov. Disord..

[53] Seidler A., Hellenbrand W., Robra B.P., Vieregge P., Nischan P., Joerg J., Oertel W.H., Ulm G., Schneider E. (1996). Possible environmental, occupational, and other etiologic factors for Parkinson’s disease: A case-control study in Germany. Neurology.

[54] Gorell J.M., Johnson C.C., Rybicki B.A., Peterson E.L., Richardson R.J. (1998). The risk of Parkinson’s disease with exposure to pesticides, farming, well water, and rural living. Neurology.

[55] Kuopio A.M., Marttila R.J., Helenius H., Rinne U.K. (1999). Environmental risk factors in Parkinson’s disease. Mov. Disord..

[56] Petrovitch H., Ross G.W., Abbott R.D., Sanderson W.T., Sharp D.S., Tanner C.M., Masaki K.H., Blanchette P.L., Popper J.S., Foley D. (2002). Plantation work and risk of Parkinson disease in a population-based longitudinal study. Arch. Neurol..

[57] Galanaud J.P., Elbaz A., Clavel J., Vidal J.S., Correze J.R., Alperovitch A., Tzourio C. (2005). Cigarette smoking and Parkinson’s disease: A case-control study in a population characterized by a high prevalence of pesticide exposure. Mov. Disord..

[58] Ascherio A., Chen H., Weisskopf M.G., O’Reilly E., McCullough M.L., Calle E.E., Schwarzschild M.A., Thun M.J. (2006). Pesticide exposure and risk for Parkinson’s disease. Ann. Neurol..

[59] Wastensson G., Hagberg S., Andersson E., Johnels B., Barregard L. (2006). Parkinson’s disease in diphenyl-exposed workers—A causal association?. Parkinsonism Relat. Disord..

[60] Kamel F., Tanner C., Umbach D., Hoppin J., Alavanja M., Blair A., Comyns K., Goldman S., Korell M., Langston J. (2007). Pesticide exposure and self-reported Parkinson’s disease in the agricultural health study. Am. J. Epidemiol..

[61] Fong C.S., Wu R.M., Shieh J.C., Chao Y.T., Fu Y.P., Kuao C.L., Cheng C.W. (2007). Pesticide exposure on southwestern Taiwanese with MnSOD and NQO1 polymorphisms is associated with increased risk of Parkinson’s disease. Clin. Chim. Acta.

[62] Brighina L., Frigerio R., Schneider N.K., Lesnick T.G., de Andrade M., Cunningham J.M., Farrer M.J., Lincoln S.J., Checkoway H., Rocca W.A. (2008). Alpha-synuclein, pesticides, and Parkinson disease: A case-control study. Neurology.

[63] Hancock D.B., Martin E.R., Mayhew G.M., Stajich J.M., Jewett R., Stacy M.A., Scott B.L., Vance J.M., Scott W.K. (2008). Pesticide exposure and risk of Parkinson’s disease: A family based case-control study. BMC Neurol..

[64] Tanner C.M., Ross G.W., Jewell S.A., Hauser R.A., Jankovic J., Factor S.A., Bressman S., Deligtisch A., Marras C., Lyons K.E. (2009). Occupation and risk of parkinsonism: A multicenter case-control study. Arch. Neurol..

[65] Elbaz A., Clavel J., Rathouz P.J., Moisan F., Galanaud J.P., Delemotte B., Alperovitch A., Tzourio C. (2009). Professional exposure to pesticides and Parkinson disease. Ann. Neurol..

[66] Dutheil F., Beaune P., Tzourio C., Loriot M.A., Elbaz A. (2010). Interaction between ABCB1 and professional exposure to organochlorine insecticides in Parkinson disease. Arch. Neurol..

[67] Firestone J.A., Lundin J.I., Powers K.M., Smith-Weller T., Franklin G.M., Swanson P.D., Longstreth W.T., Checkoway H. (2010). Occupational factors and risk of Parkinson’s disease: A population-based case-control study. Am. J. Ind. Med..

[68] Rugbjerg K., Harris M.A., Shen H., Marion S.A., Tsui J.K., Teschke K. (2011). Pesticide exposure and risk of Parkinson’s disease—A population-based case-control study evaluating the potential for recall bias. Scand. J. Work Environ. Health.

[69] Tanner C.M., Kamel F., Ross G.W., Hoppin J.A., Goldman S.M., Korell M., Marras C., Bhudhikanok G.S., Kasten M., Chade A.R. (2011). Rotenone, paraquat, and Parkinson’s disease. Environ. Health Perspect..

[70] Wang A., Costello S., Cockburn M., Zhang X., Bronstein J., Ritz B. (2011). Parkinson’s disease risk from ambient exposure to pesticides. Eur. J. Epidemiol..

[71] Feldman A.L., Johansson A.L., Nise G., Gatz M., Pedersen N.L., Wirdefeldt K. (2011). Occupational exposure in Parkinsonian disorders: A 43-year prospective cohort study in men. Parkinsonism Relat. Disord..

[72] Liew Z., Wang A., Bronstein J., Ritz B. (2014). Job exposure matrix (JEM)-derived estimates of lifetime occupational pesticide exposure and the risk of Parkinson’s disease. Arch. Environ. Occup. Health.

[73] Gunnarsson L.G., Bodin L., Soderfeldt B., Axelson O. (1992). A case-control study of motor neurone disease: Its relation to heritability, and occupational exposures, particularly to solvents. Br. J. Ind. Med..

[74] Kamel F., Umbach D.M., Munsat T.L., Shefner J.M., Hu H., Sandler D.P. (2002). Lead exposure and amyotrophic lateral sclerosis. Epidemiology.

[75] Gait R., Maginnis C., Lewis S., Pickering N., Antoniak M., Hubbard R., Lawson I., Britton J. (2003). Occupational exposure to metals and solvents and the risk of motor neuron disease. A case-control study. Neuroepidemiology.

[76] Fang F., Kwee L.C., Allen K.D., Umbach D.M., Ye W., Watson M., Keller J., Oddone E.Z., Sandler D.P., Schmidt S. (2010). Association between blood lead and the risk of amyotrophic lateral sclerosis. Am. J. Epidemiol..

[77] Graves A.B., Rosner D., Echeverria D., Mortimer J.A., Larson E.B. (1998). Occupational exposures to solvents and aluminium and estimated risk of Alzheimer’s disease. Occup. Environ. Med..

[78] Stampfer M.J. (2009). Welding occupations and mortality from Parkinson’s disease and other neurodegenerative diseases among United States men, 1985–1999. J. Occup. Environ. Hyg..

[79] Gorell J.M., Johnson C.C., Rybicki B.A., Peterson E.L., Kortsha G.X., Brown G.G., Richardson R.J. (1997). Occupational exposures to metals as risk factors for Parkinson’s disease. Neurology.

[80] Frigerio R., Elbaz A., Sanft K.R., Peterson B.J., Bower J.H., Ahlskog J.E., Grossardt B.R., de Andrade M., Maraganore D.M., Rocca W.A. (2005). Education and occupations preceding Parkinson disease: A population-based case-control study. Neurology.

[81] Coon S., Stark A., Peterson E., Gloi A., Kortsha G., Pounds J., Chettle D., Gorell J. (2006). Whole-body lifetime occupational lead exposure and risk of Parkinson’s disease. Environ. Health Perspect..

[82] Vergara X.P., Kheifets L., Silva M., Bracken T.D., Yost M. (2012). New electric-shock job exposure matrix. Am. J. Ind. Med..

[83] Fischer H., Kheifets L., Huss A., Peters T.L., Vermeulen R., Ye W., Fang F., Wiebert P., Vergara X.P., Feychting M. (2015). Occupational Exposure to Electric Shocks and Magnetic Fields and Amyotrophic Lateral Sclerosis in Sweden. Epidemiology.

[84] Fryzek J.P., Hansen J., Cohen S., Bonde J.P., Llambias M.T., Kolstad H.A., Skytthe A., Lipworth L., Blot W.J., Olsen J.H. (2005). A cohort study of Parkinson’s disease and other neurodegenerative disorders in Danish welders. J. Occup. Environ. Med..

[85] Fored C.M., Fryzek J.P., Brandt L., Nise G., Sjogren B., McLaughlin J.K., Blot W.J., Ekbom A. (2006). Parkinson’s disease and other basal ganglia or movement disorders in a large nationwide cohort of Swedish welders. Occup. Environ. Med..

[86] Kenborg L., Lassen C.F., Hansen J., Olsen J.H. (2012). Parkinson’s disease and other neurodegenerative disorders among welders: A Danish cohort study. Mov. Disord..

[87] Savitz D.A., Loomis D.P., Tse C.K. (1998). Electrical occupations and neurodegenerative disease: Analysis of U.S. mortality data. Arch. Environ. Health.

[88] Johansen C. (2000). Exposure to electromagnetic fields and risk of central nervous system disease in utility workers. Epidemiology.

[89] Weisskopf M.G., McCullough M.L., Morozova N., Calle E.E., Thun M.J., Ascherio A. (2005). Prospective study of occupation and amyotrophic lateral sclerosis mortality. Am. J. Epidemiol..

[90] Morahan J.M., Pamphlett R. (2006). Amyotrophic lateral sclerosis and exposure to environmental toxins: An Australian case-control study. Neuroepidemiology.

[91] Lindsay J., Hebert R., Rockwood K. (1997). The Canadian Study of Health and Aging: Risk factors for vascular dementia. Stroke.

