# Supplementary material for: Occupational Exposures and Neurodegenerative Diseases—A Systematic Literature Review and Meta-Analyses
Source: Int J Environ Res Public Health. 2019 Jan 26;16(3):337. doi: 10.3390/ijerph16030337 (PMC6388365; doi:10.3390/ijerph16030337)
Supplement: Supplementary file 1 [file ijerph-16-00337-s001.pdf]

Decision protocol for grading studies regarding scientific epidemiologic standard

A modified system for categorization of observational epidemiologic studies into class I-V (based on Armon [25]) was applied for cohort studies with parallel controls and case-control studies, respectively. The appendix is previously published in a recent publication [20].

Diagnosis

Cohort studies:

- Diagnosis of the studied disease made applying uniform efforts and criteria to exposed and unexposed cohorts.

Case-control studies:

- Demonstration that ascertainment of patients is complete in the given population.
- Diagnosis of the studied disease made applying established criteria.

Exposure

Cohort studies:

- Exposure, and hence assignment to the 'exposed' cohort, established before knowledge of diagnostic status, or without knowledge of diagnostic status, or confirmable independently of the knowledge of diagnostic status. Consideration of and accounting for possible misclassification.
- Unexposed cohort is appropriate to the risk factor in question, is well-matched to the exposed cohort on factors other than the exposure, and is otherwise representative of the general population.
- Exposure quantified, where possible, to permit assessment of dose-response relationships.

Case-control studies:

- Putative risk factor or exposure occurred before probable biologic onset of disease.
- Uniform effort to gather information equally from affected and unaffected individuals.
- Blinding of information-gathering method to individuals' disease status ideal; if not done – adequate justification as to why this does not affect the assessment of the risk factor in question.
- Blinding of subjects and individuals gathering the data as to the hypotheses being tested. If not done – adequate justification as to why this does not affect the assessment of the risk factor in question.
- Meticulous attention to avoiding recall bias or, if not possible, to evaluating its impact, estimating the magnitude of its impact and controlling for it.
- Exposure quantified, where possible, to permit assessment of dose-response.

Study group

Cohort studies:

- Loss to follow-up low, and comparable in exposed and unexposed cohorts. Possible roles of competing causes of mortality accounted for. Preferably – all mortality data available for both cohorts.

Case-control studies:

- Appropriate choice of controls, to assure they are matched to the patients and are also representative of the general population (assure adequate matching, avoid 'overmatching').
- High response rates from patients and controls.

Methods and Analysis

Cohort studies:

- Sources of biases and confounding identified and accounted for.
- Conclusion based on large numbers. Appropriate statistical analysis.

Case-control studies:

- Sources of biases and confounding identified and accounted for.
- Conclusions based on large numbers. Appropriate statistical analysis. Methods state if hypotheses were selected a priori for confirmatory analysis.

Criteria for categorization into Armon class

In order to form the basis of the Armon class I-V we constructed a protocol with standardized criteria for grading the categories Diagnosis, Exposure, Study group and Methods and Analysis. The protocol was graded into scores 1 to 4 as defined below. Due to lack of information sometimes a category had to be graded in between, and thus given an interval, for example 2-3.

Diagnosis: 1= the specific disease diagnosis was established by a specialist, 2=diagnosis obtained from a hospital (as an in-patient) register, 3=diagnosis from a GP (also including mortality registers), and 4= the specific disease diagnosis was not separated from other diagnoses with similar phenotype.

Exposure, Study group and Methods and Analysis: 1=good, 2=sufficient, 3=uncertain/insufficient, or 4=unacceptable.

Class I: All criteria were met within the respective design, i.e. single score 1 in every category.
Class II: Single score 1 or 2 in every category.
Class III: Single score 3 allowed for Diagnosis and 1 or 2 in the other categories (interval 2-3 is allowed).
Class IV: Single score 3 in more categories than Diagnosis.
Class V: Single score 4 in any category.
